# Extrusion and Extrusion Combined with Solid-State Enzymatic Hydrolysis Improve the Nutrient Digestion of Rapeseed Meal in Growing Pigs

**DOI:** 10.3390/ani16142132

**Published:** 2026-07-09

**Authors:** Xiaohong Guo, Jieying Jing, Aipeng Mao, Zexin Su, Shufen Xue, Jing Fu, Junning Pu, Jingyi Cai, Gang Jia, Gang Tian

**Affiliations:** Key Laboratory of Animal Disease-Resistance Nutrition of China Ministry of Education, Key Laboratory of Animal Disease-Resistant Nutrition of Sichuan Province, Animal Nutrition Institute, Sichuan Agricultural University, Chengdu 611130, China; 15392525673@163.com (X.G.); jjy2021314082@163.com (J.J.);

**Keywords:** digestibility, extrusion, growing pigs, rapeseed meal, solid-state enzymatic hydrolysis

## Abstract

Rapeseed meal (RSM) is an important protein source in animal feed, but its high levels of anti-nutritional factors and crude fiber (CF) limit its utilization in pigs. Our study compared the morphological structure, nutrient composition, and anti-nutritional factors of RSM, extruded rapeseed meal (ERM), and extruded solid-state enzymatically hydrolyzed rapeseed meal (EERM), and evaluated their digestibility in growing pigs. The results showed that ERM and EERM increased surface porosity. Notably, ERM and EERM had higher trichloroacetic acid-soluble protein contents and lower fiber, glucosinolate (GL), and isothiocyanate (ITC) contents. Compared to RSM, the in vitro digestibility of crude protein (CP) was significantly lower in ERM (*p* < 0.05), whereas EERM exhibited significantly higher digestibility of CP than ERM (*p* < 0.05). Additionally, compared to RSM and ERM, the digestibility of dry matter (DM) and gross energy (GE) increased significantly in EERM (*p* < 0.05). For in vivo digestibility, compared to ERM, the digestibility of CP and GE increased significantly in EERM (*p* < 0.05). In conclusion, our study demonstrates that ERM and EERM can change the chemical composition of RSM, and improve the digestibility of nutrients in pig production.

## Simple Summary

Rapeseed meal, the by-product of oil extraction from rapeseed, is widely used in pig feed owing to its high protein content. However, the presence of substantial crude fiber and anti-nutritional factors restricts its application in pigs. To enhance the utilization efficiency of rapeseed meal in growing pigs, we extruded and enzymatically hydrolyzed rapeseed meal, and then evaluated its nutrient absorption and utilization in growing pigs. Our study indicated that extrusion and extrusion combined with solid-state enzymatic hydrolysis can change the nutrient composition of rapeseed meal and enhance nutrient utilization in growing pigs.

## 1. Introduction

Soybean meal is the primary plant-protein source in animal feed due to its high protein and balanced amino acid (AA) profile [1,2]. However, insufficient soybean production in China and other regions has driven research interest in alternative protein sources. Rapeseed meal (RSM) is the second largest source of feed protein, containing approximately 35–45% crude protein (CP), and is rich in essential AAs such as lysine and methionine [3,4,5]. However, the presence of substantial crude fiber (CF) content and anti-nutritional factors, including glucosinolates (GLs), phytic acid, and tannins, limits its application in monogastric animal diets [6,7,8].

Numerous studies have focused on modifying RSM through extrusion [9], microbial fermentation [10,11], and enzymatic hydrolysis [12,13]. It has been reported that extrusion can reduce tannin content within a range of 20.37–41.05%, phytic acid within 20–25% [14,15], and GLs within 40–60% in RSM [16]. Moreover, extrusion can effectively inactivate myrosinase in RSM, preventing the conversion of GLs into toxic compounds and thereby enhancing palatability and safety [17]. Studies have reported that extruded rapeseed meal (ERM) improves the apparent ileal digestibility of CP in growing pigs, with increases ranging from 3.4% to 6.9% [18,19]. Additionally, replacing up to 20% of soybean meal with ERM did not reduce growth performance in the diets of weaned pigs [20]. The hydrolysis of non-starch polysaccharide (NSP) enzymes, comprising cellulase, hemicellulase, and pectinase, can release encapsulated nutrients such as proteins and starch in RSM [21,22,23]. Proteases can degrade proteins into small peptides and free AAs, thereby improving the utilization of protein [24,25]. Näsi [26] evaluated the effects of extrusion and enzymatic hydrolysis on RSM. The results showed that extrusion combined with enzymatic hydrolysis improved organic matter and protein digestibility in growing pigs. In finishing pigs, the extrusion and multienzyme preparation elevated the level of immunoglobulin G and reduced the content of malondialdehyde in serum [27]. Extrusion can increase substrate surface area but may cause thermal nutrient losses, whereas enzymatic hydrolysis alone is constrained by substrate structure. Nevertheless, the combined approach may offset the limitations, leading to improved nutrient utilization in pigs, and its potential benefits warrant investigation. In our study, we first analyzed the morphological structure and nutritional composition of RSM, ERM, and extruded solid-state enzymatically hydrolyzed rapeseed meal (EERM), and then evaluated their nutrient digestibility in growing pigs. We aim to investigate the effects of extrusion and extrusion combined with solid-state enzymatic hydrolysis on the morphological structure and nutrient composition of RSM, and to assess the digestibility of the modified rapeseed meal in growing pigs.

## 2. Materials and Methods

### 2.1. Preparation of Extruded Rapeseed Meal and Extruded Solid-State Enzymatically Hydrolyzed Rapeseed Meal

ERM was prepared from type 200 RSM using a DSE32 twin-screw extruder (Jinan Linyang Machinery Co., Ltd., Jinan, China) with a moisture content of 15%, extrusion temperature of 145 °C, spindle speed of 23 Hz, and feed rate of 10 Hz. EERM was prepared by treating ERM with the enzyme preparation under the following conditions: solid-to-liquid ratio of 1:0.99, reaction time of 9.87 h, and temperature of 46.53 °C. The composition of the multi-enzyme preparation is shown in Table S1 [28].

### 2.2. Morphological Structure and Nutrient Composition of Rapeseed Meal, Extruded Rapeseed Meal, and Extruded Solid-State Enzymatically Hydrolyzed Rapeseed Meal

#### 2.2.1. Scanning Electron Microscopy

The morphological structures of RSM, ERM, and EERM were observed using a scanning electron microscope (Sigma 360, Carl Zeiss AG, Oberkochen, Germany). Dried samples were evenly spread onto sample holders coated with conductive adhesive, and any loose particles were gently removed using a rubber suction bulb. The prepared samples were then observed and imaged under the scanning electron microscope.

#### 2.2.2. Fourier Transform Infrared Spectroscopy Analysis

For Fourier transform infrared (FTIR) spectroscopy, 2 mg of the sample was mixed with 200 mg of potassium bromide (KBr) and ground under an infrared lamp until a homogeneous mixture was obtained. The mixture was then pressed into pellets using a mold. FTIR spectra were recorded with a spectrometer (Nicolet iS10, Thermo Fisher Scientific Inc., Waltham, MA, USA), using pure KBr as the blank. Each spectrum consisted of 64 scans. Spectral resolution was improved by deconvolution using OMNIC 8.2 software (Thermo Fisher Scientific Inc., Waltham, MA, USA), and the protein secondary structure was quantitatively analyzed with Origin 8.5 software (OriginLab Corporation, Northampton, MA, USA).

#### 2.2.3. Determination of Nutrient Contents

Dry matter (DM, Method 930.15), CP (Method 954.01), CF (Method 978.10), neutral detergent fiber (NDF, Method 2002.04), acid detergent fiber (ADF, Method 973.18), acid detergent lignin (ADL, Method 973.18), ether extract (EE, Method 920.39), calcium (Ca, Method 927.02), phosphorus (P, Method 965.17), AA (Method 994.12), and tryptophan (Method 988.15) in RSM, ERM and EERM were determined according to standard procedures described by AOAC (2023) [29]. The trichloroacetic acid-soluble protein (TCA-SP) content was determined according to the method of Ovissipour et al [30].

#### 2.2.4. Determination of Glucosinolates

The content of GLs was measured using the palladium chloride method [31]. A ground sample (0.4 g) was mixed with 4 mL of distilled water and extracted in a boiling water bath for 20 min. After cooling, the mixture was filtered. Then, 2 mL of filtrate was combined with 6 mL of a solution containing 0.1% sodium carboxymethylcellulose and 1.4 mmol/L palladium chloride. Absorbance was recorded at 540 nm (A1), using sodium carboxymethylcellulose and palladium chloride as the reference. Another 2 mL of filtrate was mixed with 6 mL of 0.1% sodium carboxymethylcellulose solution, and the absorbance at 540 nm (A2) was measured, using a mixture of sodium carboxymethylcellulose and distilled water as the blank control. The content of GLs was calculated as the difference (A = A1 − A2) and quantified using a sinigrin standard curve.

#### 2.2.5. Determination of Isothiocyanate

The content of isothiocyanate (ITC) was determined following the method of Maheshwari et al [32]. A precisely weighed 0.2000 g sample was mixed with 40 mg crude myrosinase and 2.0 mL pH 7.0 buffer, then incubated at 35 °C for 2 h with intermittent vortexing. After the enzymatic reaction, 2.5 mL of dichloromethane was added, and the mixture was shaken at room temperature for 30 min, followed by centrifugation at 4000 rpm for 20 min. A 50 μL aliquot of the lower organic phase was transferred into a tube containing 6 mL of 80% ammonia ethanol. The mixture was vortexed, incubated in a 50 °C water bath for 30 min, then cooled to room temperature. Absorbance was measured at 235, 245, and 255 nm using a UV spectrophotometer, with a sample blank serving as the reference for ITC quantification.

### 2.3. Nutrient Digestibility of Rapeseed Meal, Extruded Rapeseed Meal, and Extruded Solid-State Enzymatically Hydrolyzed Rapeseed Meal

#### 2.3.1. Digestibility Experiment In Vitro

A single-factor experimental design was employed, comprising three treatment groups—RSM, ERM, and EERM—each with 4 replicates. In vitro digestion was simulated using a two-step pepsin-pancreatin method adapted from Zhu et al. [33]. Briefly, 2 g of sample was placed into a 250 mL Erlenmeyer flask, followed by the sequential addition of 100 mL phosphate buffer (0.1 mol/L, pH 6.0), 40 mL hydrochloric acid solution (0.2 mol/L), and 4 mL porcine pepsin solution (20 g/L). To inhibit bacterial contamination, 1 mL of chloramphenicol solution was added. The mixture was incubated at 39 °C with shaking at 50 rpm for 2 h. After pepsin digestion, 40 mL phosphate buffer (0.2 mol/L, pH 6.8), 20 mL sodium hydroxide solution (0.6 mol/L), and 4 mL porcine pancreatin solution (100 g/L) were added, followed by further incubation at 39 °C and 50 rpm for 4 h. Upon completion, undigested residues were sequentially washed with ethanol and acetone, filtered through a 50 μm nylon mesh, and collected for the determination of in vitro digestibility of DM, CP, and gross energy (GE). Each digestion was conducted independently for the determination of a single indicator. The content of DM and CP was determined using the methods as described previously. The content of GE was measured using an oxygen bomb calorimeter (Parr Instrument Co., Moline, IL, USA). The in vitro digestibility of nutrients was calculated using the following formula:

In vitro nutrient digestibility (%) = [(nutrient before digestion − nutrient after digestion)/nutrient before digestion] × 100.

#### 2.3.2. Digestibility Experiment In Vivo

##### Animal Experimental Design and Diets

A total of 24 crossbred barrows (Duroc × Landrace × Yorkshire) with an initial body weight of 25.60 ± 1.78 kg were randomly divided into 4 groups, each containing 6 replicates with 1 pig per replicate. Pigs were housed individually in metabolic cages (2.5 m × 1.8 m × 0.8 m) at the Teaching & Research Base, Animal Nutrition Institute of Sichuan Agricultural University. The ambient temperature was maintained at 20–25 °C. The pigs were fed at 4% of their body weight daily at 8:30 and 15:30, with free access to water.

**Figure 1:**
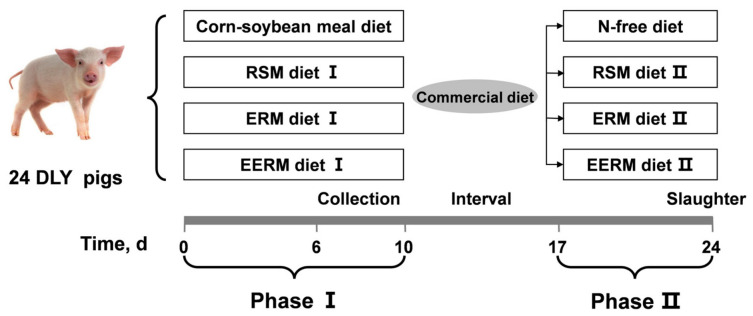
Experimental protocol.

The experiment consisted of two phases (Figure 1). Phase I (1–10 d): Pigs were fed a corn–soybean meal diet (CSD), an RSM diet I (RSD I), an ERM diet I (ERSD I), and an EERM diet I (EERSD I). Each experimental diet replaced 20% of the CSD. Diets were formulated to meet the NRC nutrient requirements proposed in 2012 for 25–50 kg growing pigs (Table S2). From 11 to 17 d, pigs were fed commercial feed and subsequently reassigned to new experimental groups. Phase II (18–24 d): Pigs were fed a N-free diet (NFD), an RSM diet II (RSD II), an ERM diet II (ERSD II), and an EERM diet II (EERSD II). Each experimental diet replaced 30% of the NFD. All diets were supplemented with 0.4% of Cr_2_O_3_ as an indigestible marker. At the end of the experiment, the pigs were stunned by electric shock and slaughtered for sample collection. The diet compositions and nutritional levels are presented in Table S3.

##### Sample Collection and Determination

Feces were collected for 4 consecutive days from 7 to 10 d (Phase I), weighed, and mixed with 10% sulfuric acid. The contents of DM, CP, and GE in diets and feces were determined using the methods as described previously. After the pigs were slaughtered (Phase II), the distal ileum was isolated and ligated, and ileal digesta samples were collected approximately 20–25 cm proximal to the ileocecal valve. The digesta samples were freeze-dried and stored for the determination of DM, AA, and Cr_2_O_3_, from which AID and SID were calculated. The contents of DM and AA in diets and digesta were determined using the same methods. Cr_2_O_3_ was determined using the method of Fenton and Fenton [34].

The calculation formula for the digestibility of nutrients is as follows:

Apparent digestibility (%) = [(nutrient intake − nutrient in feces)/nutrient intake] × 100

The calculation formula for the digestibility of the ingredient is as follows:

D_nutrient_ = [D_test_ − (1 − X) × D_CSD_]/X × 100

where D_nutrient_ is the digestibility of the nutrient in the test ingredient (%), D_test_ is the digestibility of the nutrient in the test diet (%), D_CSD_ is the digestibility of the nutrient in the CSD (%), and X is the substitution ratio (proportion of the test ingredient replacing the basal diet in the test diet).

The calculation formulas for AID and SID are as follows:

AID (%) = [1 − (Cr_2_O_3_ in diet/Cr_2_O_3_ in digesta) × (AA in digesta/AA in diet)] × 100

SID (%) = AID + [(Cr_2_O_3_ in N-free diet/Cr_2_O_3_ in N-free diet digesta) × (AA in N-free diet digesta/AA in diet) × 100].

### 2.4. Statistical Analysis

The results are presented as means ± standard error of the mean (SEM). All data were analyzed using one-way analysis of variance (ANOVA) with SPSS 27.0 software (SPSS Inc., Chicago, IL, USA). Duncan’s multiple range test was used for post hoc comparisons. Statistical significance was defined at *p* < 0.05. GraphPad Prism 10.1.2 software (GraphPad Software, San Diego, CA, USA) was used for graphing.

## 3. Results

### 3.1. Morphological Structure and Nutrient Composition of Rapeseed Meal, Extruded Rapeseed Meal, and Extruded Solid-State Enzymatically Hydrolyzed Rapeseed Meal

**Figure 2:**
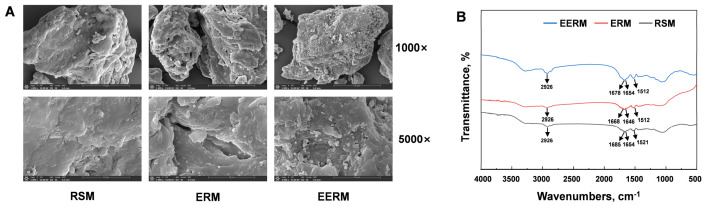
Morphological structure of rapeseed meal, extruded rapeseed meal, and extruded solid-state enzymatically hydrolyzed rapeseed meal. (**A**) Scanning electron microscopy micrographs. (**B**) Fourier transform infrared spectra. RSM, rapeseed meal; ERM, extruded rapeseed meal; EERM, extruded solid-state enzymatically hydrolyzed rapeseed meal.

Scanning electron microscopy (Figure 2A) revealed that RSM possessed a compact and smooth surface with no visible pores, whereas ERM and EERM exhibited looser and more porous surface structures. Notably, more small granular structures were observed on the surface of EERM. FTIR spectroscopy results (Figure 2B) showed that, compared with RSM, the absorption peak of ERM shifted from 1685 cm^−1^ to 1668 cm^−1^, indicating an increase in β-turn. The absorption peak of EERM decreased at 1654 cm^−1^, suggesting a reduction in α-helix content. Both extrusion and solid-state enzymatic hydrolysis treatments changed the C=O bonds of proteins. Compared with ERM, the absorption peak of EERM at 2926 cm^−1^ was enhanced, indicating an increase in reducing sugar (2800–3000 cm^−1^) content and changes in C–H bonds.

The GE values of RSM, ERM, and EERM were 18.97 MJ/kg, 19.42 MJ/kg, and 18.49 MJ/kg, respectively. Compared to RSM (Table 1), the contents of NDF and P are higher in ERM, and the content of ADF is lower. The contents of CP, CF, NDF, ADF, and ADL are lower in EERM. Compared to ERM, the contents of CP, CF, NDF, ADF, and ADL are lower in EERM. The content of TCA-SP was highest in EERM (14.61%), followed by ERM (2.83%), and lowest in RSM (2.23%). The content of GLs was highest in RSM (61.51 μmol/g), intermediate in ERM (29.95 μmol/g), and lowest in EERM (16.07 μmol/g). The content of ITC was highest in RSM (2.22 mg/g), intermediate in ERM (0.30 mg/g), and not detected in EERM. in EERM. Regarding AA composition (Table 2), compared to RSM, the content of Met is higher in ERM, and the contents of Lys, Thr, Ile, Arg, Asp, Glu, Gly, Cys, and total amino acids (TAA) are lower. The content of Met is higher in EERM, and the contents of Lys, Thr, His, Arg, Asp, Ser, Glu, Gly, Cys, and TAA are lower in EERM. Compared to ERM, the contents of Thr, His, Arg, Glu, and TAA are lower.

### 3.2. Nutrient Digestibility of Rapeseed Meal, Extruded Rapeseed Meal, and Extruded Solid-State Enzymatically Hydrolyzed Rapeseed Meal in Growing Pigs

**Figure 3:**
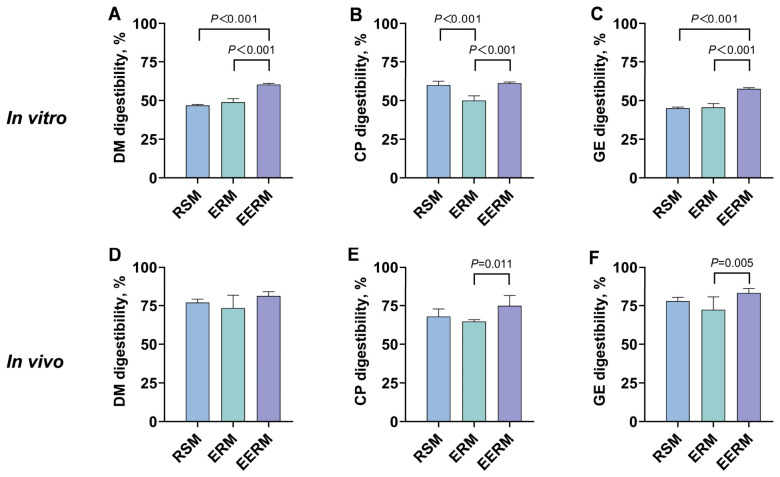
The digestibility of rapeseed meal, extruded rapeseed meal, and extruded solid-state enzymatically hydrolyzed rapeseed meal in vitro and in vivo (DM basis, %). (**A**–**C**) The digestibility of DM, CP, and GE in vitro. (**D**–**F**) The digestibility of DM, CP, and GE in vivo. RSM, rapeseed meal; ERM, extruded rapeseed meal; EERM, extruded solid-state enzymatically hydrolyzed rapeseed meal. DM, dry matter; CP, crude protein; GE, gross energy.

As shown in Figure 3A–C, compared to RSM, the digestibility of CP decreased significantly in ERM (*p* < 0.05), and the digestibility of DM and GE increased significantly in EERM (*p* < 0.05). Compared to ERM, the digestibility of DM, CP, and GE increased significantly in EERM (*p* < 0.05). As shown in Figure 3D–F, compared to ERM, the digestibility of CP and GE increased significantly in EERM (*p* < 0.05). The AID and SID of all AA in RSM, ERM, and EERM did not differ significantly (*p* > 0.05), as presented in Table 3 and Table 4.

## 4. Discussion

The application of RSM in animal diets is limited due to its anti-nutritional factors and CF content. In our study, we compared the morphological structure and nutrient composition of RSM, ERM, and EERM. The results demonstrated that extrusion disrupted the dense structure of RSM, making it into a loose and porous matrix. Treatment with the multi-enzyme preparation further increased the surface porosity and produced finer particles. These structural modifications increased the contact area between enzymes and substrates, promoting the enzymatic hydrolysis efficiency of RSM. FTIR analysis revealed an increase in β-turn content in ERM and a reduction in α-helix content in EERM, with lower α-helix levels, indicating partial protein denaturation or structural modification.

Regarding nutrient composition, the contents of CP and GE in EERM were lower than those in ERM, suggesting that the processing compromises the nutrients in RSM. The contents of CF, NDF, ADF, and ADL were reduced in EERM. Previous studies have shown that supplementing RSM with cellulase or applying fermentation can decrease fiber fractions [7,35]. Fiber impedes the digestion and absorption of proteins and carbohydrates [36]. The addition of NSP enzymes promotes the degradation and restructuring of macromolecules such as cellulose and hemicellulose, thereby reducing fiber content [37]. The fiber degradation not only enhances the digestibility of energy and nutrients by releasing more soluble sugars and improving nutritional value but also shortens the retention time of digesta in the hindgut, ultimately improving feed conversion efficiency [38,39]. Compared with RSM, the TCA-SP content increased modestly from 2.23% to 2.83% after extrusion, and further to 14.61% after subsequent enzymatic hydrolysis, indicating that extrusion combined with solid-state enzymatic hydrolysis converted proteins into small peptides and free AAs. Previous studies that utilized extrusion pretreatment followed by microbial fermentation reported a marked increase in the small-peptide (<3 kDa) content in RSM [40]. Small peptides and free AAs are rapidly absorbed by porcine intestinal epithelial cells, enhancing nitrogen retention efficiency and providing a more readily available nitrogen source for the animal [41]. Extrusion effectively reduced the GLs and ITC in RSM [42,43]. In the present study, extrusion combined with solid-state enzymatic hydrolysis further decreased the content of GLs and ITC in EERM, indicating that the combined treatment is more effective in reducing these anti-nutritional factors than extrusion alone [44]. Consistent with the observed reduction in CP content, after extrusion and extrusion combined with solid-state enzymatic hydrolysis of RSM, all AAs except Met decreased, possibly due to Maillard reactions between reducing sugars and AAs at high extrusion temperatures [45,46]. Lys is the first limiting AA in cereal-based diets for pigs. This reduction in available Lys may compromise protein utilization, suggesting that Lys supplementation is recommended when ERM is included in the diet [47,48].

Digestibility is a crucial parameter for evaluating the nutritional value and productive performance of feed ingredients. We evaluated in vitro and in vivo digestibility of DM, CP, and GE to assess the effects of extrusion and extrusion combined with solid-state enzymatic hydrolysis on RSM modification. The in vitro CP digestibility of ERM decreased, whereas the in vitro digestibility of DM and GE increased in EERM, with EERM demonstrating superior effects compared to ERM. Regarding in vivo digestibility, EERM exhibited higher digestibility of CP and GE than ERM. The decrease in CP digestibility after extrusion may be attributed to heat-induced changes in protein structure or a reduction in relative protein content [49]. The multi-enzyme preparation reduced the fiber fractions in EERM, and since fiber content is negatively correlated with energy digestibility [50], the GE digestibility of EERM increased. Solid-state enzymatic hydrolysis also reduced NSP and antinutritional factors, which pigs cannot digest endogenously and that interfere with nutrient digestion, absorption, and utilization [51,52]. Their reduction thus facilitated improved digestibility of DM, CP, and GE. Improved CP digestibility promotes protein absorption [53,54].

In the present study, no significant differences in AID or SID of AA were observed among RSM, ERM, and EERM. A previous study optimizing enzymatic hydrolysis of hemp seed protein demonstrated that protein unfolding increased enzyme-substrate contact by exposing aromatic AA residues in the hydrophobic core, improving the digestibility of Tyr and Phe [55]. That study indicated that moderate enzymatic hydrolysis can disrupt protein conformation, making previously buried AA residues accessible and enhancing digestibility [55]. However, such effects were not observed under our experimental conditions. Another study reported that dietary supplementation with exogenous proteases significantly increased SID of limiting AA, such as Lys, Met, and Thr, in growing pigs, with improvements depending on protease type and dosage [56]. Nevertheless, that study used purified protease, whereas we employed a multi-enzyme preparation with relatively low protease activity. Moreover, the contents of Lys and Met in RSM are inherently low and tend to form complexes with anti-nutritional factors such as phytic acid and tannins, limiting enzymatic release efficiency [57].

## 5. Conclusions

In summary, extrusion and extrusion combined with enzymatic hydrolysis changed the morphological structure and nutrient composition of RSM, contributing to improved digestibility of CP and GE. However, both extrusion and extrusion combined with solid-state enzymatic hydrolysis decreased most AAs except Met, which should be considered in practical applications.

## Figures and Tables

**Table 1:** Nutrient composition and anti-nutritional factors of rapeseed meal, extruded rapeseed meal, and extruded solid-state enzymatically hydrolyzed rapeseed meal (DM basis, %).

Items	RSM	ERM	EERM
CP	41.78	40.75	39.29
CF	12.55	13.09	10.21
NDF	35.19	49.19	22.54
ADF	25.75	23.27	18.45
ADL	17.99	17.69	14.37
EE	1.33	1.32	1.65
Ca	0.90	0.99	0.98
P	1.25	1.31	1.23
TCA-SP	2.23	2.83	14.61
GLs, μmol/g	61.51	29.95	16.07
ITC, mg/g	2.22	0.30	0

RSM, rapeseed meal; ERM, extruded rapeseed meal; EERM, extruded solid-state enzymatically hydrolyzed rapeseed meal; CP, crude protein; CF, crude fiber; NDF, neutral detergent fiber; ADF, acid detergent fiber; ADL, acid detergent lignin; EE, ether extract; Ca, calcium; P, phosphorus; TCA-SP, trichloroacetic acid-soluble protein; GLs, glucosinolates; ITC, isothiocyanate.

**Table 2:** The content of amino acids in rapeseed meal, extruded rapeseed meal, and extruded solid-state enzymatically hydrolyzed rapeseed meal (DM basis, %).

Items	RSM	ERM	EERM
Essential AA			
Lys	2.31	1.60	1.55
Met	0.66	0.71	0.72
Thr	1.75	1.56	1.48
Trp	0.49	0.46	0.47
Val	2.14	1.99	2.04
Ile	1.61	1.46	1.51
Leu	2.55	2.48	2.47
Phe	1.54	1.46	1.46
His	1.25	1.23	1.15
Arg	2.16	1.98	1.84
Non-essential AA			
Asp	2.75	2.51	2.51
Ser	1.44	1.30	1.15
Glu	7.15	6.78	6.58
Gly	2.03	1.87	1.87
Ala	1.63	1.74	1.62
Cys	0.42	0.35	0.37
Tyr	0.58	0.51	0.53
Pro	2.95	3.03	2.80
TAA	35.43	33.03	32.12

RSM, rapeseed meal; ERM, extruded rapeseed meal; EERM, extruded solid-state enzymatically hydrolyzed rapeseed meal; AA, amino acids; TAA, total amino acids.

**Table 3:** The apparent ileal digestibility of amino acids in rapeseed meal, extruded rapeseed meal, and extruded solid-state enzymatically hydrolyzed rapeseed meal in growing pigs (DM basis, %).

Items	RSM	ERM	EERM	*p*-Value
Essential AA				
Lys	52.29 ± 3.94	52.93 ± 7.71	46.97 ± 5.18	0.710
Met	30.80 ± 5.51	44.18 ± 3.17	49.16 ± 7.31	0.091
Thr	52.36 ± 3.38	59.77 ± 2.32	51.96 ± 4.55	0.362
Trp	49.68 ± 4.47	59.17 ± 3.52	53.04 ± 4.46	0.378
Val	59.75 ± 2.89	67.67 ± 2.61	57.83 ± 4.49	0.219
Ile	61.37 ± 2.78	67.95 ± 2.54	58.54 ± 4.58	0.261
Leu	64.08 ± 2.82	69.82 ± 2.43	60.01 ± 4.57	0.238
Phe	66.81 ± 2.45	72.11 ± 2.79	67.82 ± 3.70	0.521
His	70.92 ± 2.54	71.27 ± 5.18	63.67 ± 3.93	0.315
Arg	71.18 ± 2.59	70.74 ± 4.28	66.18 ± 3.95	0.558
Non-essential AA				
Asp	52.04 ± 4.15	54.49 ± 7.48	54.12 ± 4.08	0.937
Ser	58.45 ± 3.56	63.16 ± 1.39	58.98 ± 4.22	0.667
Glu	75.27 ± 2.34	75.05 ± 4.94	71.01 ± 2.68	0.592
Gly	50.83 ± 5.88	41.62 ± 12.62	44.73 ± 7.91	0.733
Ala	58.46 ± 3.20	62.91 ± 2.20	56.70 ± 4.93	0.583
Cys	73.36 ± 2.45	61.64 ± 9.84	72.44 ± 0.59	0.329
Tyr	56.41 ± 3.35	69.02 ± 1.17	58.38 ± 4.23	0.082
Pro	51.94 ± 3.84	33.71 ± 14.04	53.02 ± 2.98	0.235
TAA	62.05 ± 3.10	58.92 ± 6.36	58.25 ± 4.84	0.830

RSM, rapeseed meal; ERM, extruded rapeseed meal; EERM, extruded solid-state enzymatically hydrolyzed rapeseed meal; AA, amino acids; TAA, total amino acids.

**Table 4:** The standardized ileal digestibility of amino acids in rapeseed meal, extruded rapeseed meal, and extruded solid-state enzymatically hydrolyzed rapeseed meal in growing pigs (DM basis, %).

Items	RSM	ERM	EERM	*p*-Value
Essential AA				
Lys	60.90 ± 3.94	62.94 ± 7.71	57.59 ± 5.18	0.797
Met	42.26 ± 5.51	58.45 ± 3.17	63.29 ± 7.31	0.048
Thr	63.19 ± 3.38	72.30 ± 2.32	63.75 ± 4.55	0.263
Trp	61.23 ± 4.47	71.60 ± 3.52	65.09 ± 4.45	0.319
Val	66.77 ± 2.89	75.29 ± 2.61	65.09 ± 4.49	0.192
Ile	68.84 ± 2.78	70.51 ± 6.06	66.26 ± 4.58	0.803
Leu	71.27 ± 2.82	77.81 ± 2.43	67.78 ± 4.57	0.224
Phe	74.85 ± 2.45	80.81 ± 2.79	76.10 ± 3.70	0.446
His	76.61 ± 2.54	77.40 ± 5.18	69.68 ± 3.93	0.324
Arg	77.85 ± 2.59	78.37 ± 4.28	74.08 ± 3.95	0.661
Non-essential AA				
Asp	62.23 ± 4.15	65.76 ± 7.48	64.71 ± 4.08	0.887
Ser	68.33 ± 3.56	75.31 ± 1.38	70.18 ± 4.22	0.457
Glu	79.83 ± 2.34	79.93 ± 4.94	75.69 ± 2.68	0.590
Gly	73.23 ± 5.88	65.95 ± 12.62	67.91 ± 7.91	0.814
Ala	67.87 ± 3.20	73.53 ± 2.20	66.37 ± 4.93	0.482
Cys	83.50 ± 2.45	72.27 ± 9.84	82.97 ± 0.59	0.350
Tyr	64.79 ± 3.35	77.02 ± 1.17	66.09 ± 4.23	0.086
Pro	75.17 ± 3.84	56.83 ± 14.04	73.91 ± 2.98	0.257
TAA	66.80 ± 3.10	64.11 ± 6.36	63.22 ± 4.84	0.853

RSM, rapeseed meal; ERM, extruded rapeseed meal; EERM, extruded solid-state enzymatically hydrolyzed rapeseed meal; AA, amino acids; TAA, total amino acids.

## Data Availability

The data that support the findings of this study are available from the corresponding author upon reasonable request.

## Supplementary Materials

The following supporting information can be downloaded at: https://www.mdpi.com/article/10.3390/ani16142132/s1, Table S1: Composition of the multi-enzyme preparation. Table S2: Ingredient composition and nutritional level of phase I (as-fed basis, %). Table S3: Ingredient composition and nutritional level of phase II (as-fed basis, %).

## Abbreviations

The following abbreviations are used in this manuscript:

ADF	Acid detergent fiber
ADL	Acid detergent lignin
AA	Amino acids
AID	Apparent ileal digestibility
CP	Crude protein
CF	Crude fiber
Ca	Calcium
DM	Dry matter
EE	Ether extract
ERM	Extruded rapeseed meal
EERM	Extruded solid-state enzymatically hydrolyzed rapeseed meal
FTIR	Fourier transform infrared
GE	Gross energy
GLs	Glucosinolates
ITC	Isothiocyanate
KBr	Potassium bromide
NDF	Neutral detergent fiber
NSP	Non-starch polysaccharide
P	Phosphorus
RSM	Rapeseed meal
SID	Standardized ileal digestibility
TAA	Total amino acids
TCA-SP	Trichloroacetic acid-soluble protein

## References

[1] Eklund M., Sauer N., Rink F., Mademacher R., Mosenthin R. (2012). Effect of Soybean Meal Origin on Standardized Ileal Amino Acid Digestibility in Piglets. J. Anim. Sci..

[2] Mukherjee R., Chakraborty R., Dutta A. (2015). Role of Fermentation in Improving Nutritional Quality of Soybean Meal—A Review. Asian-Australas. J. Anim. Sci..

[3] Slominski B.A., Jia W., Rogiewicz A., Nyachoti C.M., Hickling D. (2012). Low-Fiber Canola. Part 1. Chemical and Nutritive Composition of the Meal. J. Agric. Food Chem..

[4] Mejicanos G., Sanjayan N., Kim I.H., Nyachoti C.M. (2016). Recent Advances in Canola Meal Utilization in Swine Nutrition. J. Anim. Sci. Technol..

[5] Long C., Rösch C., De Vries S., Schols H., Venema K. (2020). Cellulase and Alkaline Treatment Improve Intestinal Microbial Degradation of Recalcitrant Fibers of Rapeseed Meal in Pigs. J. Agric. Food Chem..

[6] Shi C., He J., Yu J., Yu B., Mao X., Zheng P., Huang Z., Chen D. (2015). Amino Acid, Phosphorus, and Energy Digestibility of Aspergillus Niger Fermented Rapeseed Meal Fed to Growing Pigs. J. Anim. Sci..

[7] Navarro D.M.D., Liu Y., Bruun T., Stein H.H. (2017). Amino Acid Digestibility by Weanling Pigs of Processed Ingredients Originating from Soybeans, 00-Rapeseeds, or a Fermented Mixture of Plant Ingredients. J. Anim. Sci..

[8] Ullah Z., Rehman Z.U., Yin Y., Stein H.H., Hayat Z., Ahmed G., Nisa M.U., Akhtar M., Sarwar Z. (2017). Comparative Ileal Digestibility of Amino Acids in 00-Rapeseed Meal and Rapeseed Meal Fed to Growing Male Broilers. Poult. Sci..

[9] Cheng H., Liu X., Xiao Q., Zhang F., Liu N., Tang L., Wang J., Ma X., Tan B., Chen J. (2022). Rapeseed Meal and Its Application in Pig Diet: A Review. Agriculture.

[10] Deng H., Jian X., Li L., Zhou Q., Pu Y., Wang F., Zeng J., Su Y. (2025). Solid-State Fermentation of Rapeseed Meal Using Schizochytrium ATCC 20888 to Improve Docosahexaenoic Acid and Degradate Toxin. Food Biosci..

[11] Zhu X., Chen Y., Hao S., Jin S., Li X. (2023). Improvement of the Nutritional Quality of Rapeseed Meal through Solid-State Fermentation with *B. Subtilis*, *S. Cerevisiae*, and *B. Amyloliquefaciens*. Fermentation.

[12] Du Z., Sang N., Yu B., Zheng P., Wang H., Wang Q., Chen D. (2025). Obtaining Higher Yield and Quality Rapeseed Protein: Complex Enzymatic Hydrolysis Assisted Alkaline Water Extraction of Cold Pressed-Extracted Rapeseed Meal. Food Chem. X.

[13] Du Z., Chen D., Sang N., Wang H., Wang Q., Zheng P., Yu B. (2025). Preparation of Rapeseed Protein by Enzymatic Hydrolysis Assisted Alkaline Water Extraction and Its Nutritional Value in Comparison with Rapeseed Meal and Soybean Protein Concentrate. LWT.

[14] Han R., McDowell R., Gaunt S., Mondor M., Hernández-Álvarez A.J. (2025). Transforming Oilseed Blends: The Impact of Low-Moisture Extrusion on Antinutritional Factors, Protein Structure, and Nutritional Value. Food Chem..

[15] Rodrigues I.M., Carvalho M.G.V., Rocha J.M. (2017). Increase of Protein Extraction Yield from Rapeseed Meal through a Pretreatment with Phytase. J. Sci. Food Agric..

[16] Huang S., Liang M., Lardy G., Huff H.E., Kerley M.S., Hsieh F. (1995). Extrusion Processing of Rapeseed Meal for Reducing Glucosinolates. Anim. Feed Sci. Technol..

[17] Maskell I., Ellis M., Smithard R. (1988). Nutritive Value of Pig Diets Containing Extruded or Milled Full Fat Rapeseed. Proc. Br. Soc. Anim. Prod..

[18] Heyer C.M.E., Wang L.F., Beltranena E., Zijlstra R.T. (2021). Nutrient Digestibility of Extruded Canola Meal in Ileal-Cannulated Growing Pigs and Effects of Its Feeding on Diet Nutrient Digestibility and Growth Performance in Weaned Pigs. J. Anim. Sci..

[19] Salazar-Villanea S., Bruininx E.M.A.M., Gruppen H., Hendriks W.H., Carré P., Quinsac A., Van Der Poel A.F.B. (2018). Pelleting and Extrusion Can Ameliorate Negative Effects of Toasting of Rapeseed Meal on Protein Digestibility in Growing Pigs. Animal.

[20] Landero J.L., Beltranena E., Cervantes M., Araiza A.B., Zijlstra R.T. (2012). The effect of feeding expeller-pressed canola meal on growth performance and diet nutrient digestibility in weaned pigs. Anim. Feed Sci. Technol..

[21] Long C., Venema K. (2020). Pretreatment of Rapeseed Meal Increases Its Recalcitrant Fiber Fermentation and Alters the Microbial Community in an *in Vitro* Model of Swine Large Intestine. Front. Microbiol..

[22] Rommi K., Hakala T.K., Holopainen U., Nordlund E., Poutanen K., Lantto R. (2014). Effect of Enzyme-Aided Cell Wall Disintegration on Protein Extractability from Intact and Dehulled Rapeseed (*Brassica Rapa* L. and *Brassica Napus* L.) Press Cakes. J. Agric. Food Chem..

[23] Pedersen N.R., Ravn J.L., Pettersson D. (2017). A Multienzyme NSP Product Solubilises and Degrades NSP Structures in Canola and Mediates Protein Solubilisation and Degradation in Vitro. Anim. Feed Sci. Technol..

[24] de Oliveira Sousa T., Araújo da Silva N., de Melo Oliveira V., da Silva Ramos A.V., Barbosa Filho J.P.M., Batista J.M.d.S., Brandão Costa R.M.P., Porto A.L.F., Bezerra Pinheiro de Lima S., de Paula Ferreira Teixeira M. (2025). Use of Proteases for Animal Feed Supplementation: Scientific and Technological Updates. Prep. Biochem. Biotechnol..

[25] Wang Y., Sun H., Liu X. (2022). A Novel Fermented Rapeseed Meal, Inoculated with Selected Protease-Assisting Screened *B. Subtilis* YY-4 and *L. Plantarum* 6026, Showed High Availability and Strong Antioxidant and Immunomodulation Potential Capacity. Foods.

[26] Näsi M. (1991). Digestibility and Protein Utilization Responses of Soybean and Rape Seed Meal to Physical and Enzymatic Treatments in Diets for Growing Pigs. Agric. Food Sci..

[27] Xie P., Huang H.L., Dong X.Y., Zou X.T. (2012). Evaluation of Extruded or Unextruded Double-Low Rapeseed Meal and Multienzymes Preparation in Pigs Nutrition during the Finishing Phase of Production. Ital. J. Anim. Sci..

[28] Jing J. (2024). Development of Extruded-Solid-State Enzymatic Hydrolysis of Rapeseed Meal and Its Nutritional Value Evaluation in Growing Pigs. Master’s Thesis.

[29] AOAC (2023). Official Methods of Analysis of AOAC International.

[30] Ovissipour M., Abedian A., Motamedzadegan A., Rasco B., Safari R., Shahiri H. (2009). The Effect of Enzymatic Hydrolysis Time and Temperature on the Properties of Protein Hydrolysates from Persian Sturgeon (*Acipenser persicus*) Viscera. Food Chem..

[31] Wathelet J.-P., Wagstaffe P.J., Biston R., Marlier M., Severin M. (1988). Rapeseed Reference Materials for Glucosinolate Analysis: Development of Rapeseed BCR RM 190 and the Results of the Intercomparison of Methods. Z. Anal. Chem..

[32] Mukhopadhyay S., Bhattacharyya D.K. (1983). Colorimetric Estimation of Allyl Isothiocyanate Content in Mustard and Rapeseed Oils. Fette Seifen Anstrichm..

[33] Zhu X., Wang L., Zhang Z., Ding L., Hang S. (2021). Combination of Fiber-Degrading Enzymatic Hydrolysis and Lactobacilli Fermentation Enhances Utilization of Fiber and Protein in Rapeseed Meal as Revealed in Simulated Pig Digestion and Fermentation in Vitro. Anim. Feed Sci. Technol..

[34] Fenton T.W., Fenton M. (1979). An Improved Procedure for the Determination of Chromic Oxide in Feed and Feces. Can. J. Anim. Sci..

[35] Shuai C., Chen D., Yu B., Luo Y., Zheng P., Huang Z., Yu J., Mao X., Yan H., He J. (2023). Effect of Fermented Rapeseed Meal on Growth Performance, Nutrient Digestibility, and Intestinal Health in Growing Pigs. Anim. Nutr..

[36] Zhang S., de Vries S., Gerrits W.J.J. (2024). Quantifying the Effects of Dietary Fibres on Protein Digestibility in Pigs: A Review. Anim. Feed Sci. Technol..

[37] Yang Z., Huang Z., Cao L. (2022). Biotransformation Technology and High-Value Application of Rapeseed Meal: A Review. Bioresour. Bioprocess..

[38] Cheng Y.-H., Su L.-W., Horng Y.-B., Yu Y.-H. (2019). Effects of Soybean Meal Fermented by Lactobacillus Species and Clostridium Butyricum on Growth Performance, Diarrhea Incidence, and Fecal Bacteria in Weaning Piglets. Ann. Anim. Sci..

[39] Li P., Wang F., Wu F., Wang J., Liu L., Lai C. (2015). Chemical Composition, Energy and Amino Acid Digestibility in Double-Low Rapeseed Meal Fed to Growing Pigs. J. Anim. Sci. Biotechnol..

[40] Wang Y., Sun H., Han B., Li H.Y., Liu X.L. (2022). Improvement of Nutritional Value, Molecular Weight Patterns (Soluble Peptides), Free Amino Acid Patterns, Total Phenolics and Antioxidant Activity of Fermented Extrusion Pretreatment Rapeseed Meal with *Bacillus subtilis* YY-1 and *Saccharomyces cerevisiae* YY-2. LWT.

[41] Nosworthy M.G., Bertolo R.F., Brunton J.A. (2013). Ontogeny of Dipeptide Uptake and Peptide Transporter 1 (PepT1) Expression along the Gastrointestinal Tract in the Neonatal Yucatan Miniature Pig. Br. J. Nutr..

[42] Liang M., Huang S., Huff H.E., Kerley M.S., Hsieh F. (2002). Extrusion Cooking of Rapeseed Meal for Feeding Value Improvement. Appl. Eng. Agric..

[43] Sakac M.B., Filipovic S.S., Borojevic C.M., Ristic M.D., Kormanjos S.M. (2006). The Influence of Extrusion on Total Glucosinolates and Total Phenols in Rapeseed. Acta Agric. Serb..

[44] Zhang Z., Li P., Liu L., Zhang S., Li J., Zhang L., Li D. (2020). Ether Extract and Acid Detergent Fibre but Not Glucosinolates Are Determinants of the Digestible and Metabolizable Energy of Rapeseed Meal in Growing Pigs. J. Appl. Anim. Res..

[45] Recoules E., Lessire M., Labas V., Duclos M.J., Soia L.C., Lardic L., Peyronnet C., Quinsac A., Narcy A., Réhault-Godbert S. (2019). Digestion Dynamics in Broilers Fed Rapeseed Meal. Sci. Rep..

[46] Ghazalah A.A., El-Kaiaty A.M., Motawe H.F.A., Radwan A.S. (2020). Nutritional Impact of Canola Meal on Performance, Blood Constituents and Immune Response of Broilers. J. Agric. Sci..

[47] Hasan M.S., Crenshaw M.A., Liao S.F. (2020). Dietary Lysine Affects Amino Acid Metabolism and Growth Performance, Which May Not Involve the GH/IGF-1 Axis, in Young Growing Pigs. J. Anim. Sci..

[48] Chang Y.M., Wei H.W. (2005). The Effects of Dietary Lysine Deficiency on Muscle Protein Turnover in Postweanling Pigs. Asian-Australas. J. Anim. Sci..

[49] Lee J.W., Patterson R., Woyengo T.A. (2018). Porcine *in Vitro* Degradation and Fermentation Characteristics of Canola Co-Products without or with Fiber-Degrading Enzymes. Anim. Feed Sci. Technol..

[50] Huang Q., Piao X.S., Ren P., Li D.F. (2012). Prediction of Digestible and Metabolizable Energy Content and Standardized Ileal Amino Acid Digestibility in Wheat Shorts and Red Dog for Growing Pigs. Asian-Australas. J. Anim. Sci..

[51] Aranda-Aguirre E., Robles-Jimenez L.E., Osorio-Avalos J., Vargas-Bello-Pérez E., Gonzalez-Ronquillo M. (2021). A Systematic-Review on the Role of Exogenous Enzymes on the Productive Performance at Weaning, Growing and Finishing in Pigs. Vet. Anim. Sci..

[52] Choct M., Dersjant-Li Y., McLeish J., Peisker M. (2010). Soy Oligosaccharides and Soluble Non-Starch Polysaccharides: A Review of Digestion, Nutritive and Anti-Nutritive Effects in Pigs and Poultry. Asian-Australas. J. Anim. Sci..

[53] Tang J., Li W., Zhou Q., Fang Z., Lin Y., Xu S., Feng B., Zhuo Y., Jiang X., Zhao H. (2023). Effect of Heating, Microbial Fermentation, and Enzymatic Hydrolysis of Soybean Meal on Growth Performance, Nutrient Digestibility, and Intestinal Microbiota of Weaned Piglets. J. Anim. Sci..

[54] Ansia I., Stein H.H., Brøkner C., Hayes C.A., Drackley J.K. (2021). Nutrient Digestibility and Endogenous Protein Losses in the Foregut and Small Intestine of Weaned Dairy Calves Fed Calf Starters with Conventional or Enzyme-Treated Soybean Meal. J. Dairy Sci..

[55] Baek I., Jang Y., Lee W. (2025). Optimization of Hydrolysis Conditions to Enhance Solubility of Hempseed (*Cannabis Sativa* L.) Protein Isolate Using Response Surface Methodology for Functional Beverage Application. Food Sci. Preserv..

[56] Galli G.M., Levesque C.L., Cantarelli V.S., Chaves R.F., Silva C.C., Fascina V.B., Perez-Palencia J.Y. (2024). Effect of Protease Supplementation on Amino Acid Digestibility of Soybean Meal Fed to Growing-Finishing Pigs in Two Different Ages. J. Anim. Sci..

[57] Khajali F., Slominski B.A. (2012). Factors That Affect the Nutritive Value of Canola Meal for Poultry. Poult. Sci..

