# Supplementary material for: Extrusion and Extrusion Combined with Solid-State Enzymatic Hydrolysis Improve the Nutrient Digestion of Rapeseed Meal in Growing Pigs
Source: Animals (Basel). 2026 Jul 9;16(14):2132. doi: 10.3390/ani16142132 (PMC13405796; doi:10.3390/ani16142132)
Supplement: Supplementary file 1 [file animals-16-02132-s001.zip › animals-4397656-supplementary.pdf]

Table S1. Composition of the multi-enzyme preparation.

Enzyme types	Appending proportion, %	Enzyme activity, U/g
Cellulase	0.3	≥8000
Pectinase	0.5	≥25,000
Xylanase	0.1	≥8000
Mannanase	0.5	≥14,000
β-glucanase	0.3	≥50,000
Acid protease	0.1	≥9000
Neutral protease	0.4	≥50,000
Alkaline protease	0.4	≥200,000
Papain	0.5	≥35,000

Table S2. Ingredient composition and nutritional level of phase Ⅰ (as-fed basis, %)

Item, %	CSD	RSD I	ERSD I	EERSD I
Ingredient composition				
Corn	74.27	59.42	59.42	59.42
Soybean meal	23.00	18.40	18.40	18.40
RSM	0.00	19.30	0.00	0.00
ERM	0.00	0.00	19.32	0.00
EERM	0.00	0.00	0.00	19.42
Choline chloride (50%)	0.15	0.15	0.15	0.15
$CaCO_3$	0.25	0.40	0.38	0.28
$CaHPO_4$	1.50	1.50	1.50	1.50
NaCl	0.50	0.50	0.50	0.50
Vitamin premix[1]	0.03	0.03	0.03	0.03
Mineral premix[2]	0.30	0.30	0.30	0.30
Total	100.00	100.00	100.00	100.00
Analyzed composition[3]				
Dry matter	87.28	87.35	87.66	88.41
Gross energy, MJ/kg	15.60	15.74	15.81	15.94
Crude protein	14.83	19.58	18.93	19.97
Crude fiber	2.84	4.93	4.78	4.63
Ether Extract	2.15	2.01	2.10	1.96
Ash	4.35	5.13	5.21	5.51

[1] Vitamin premix provided the following per kg of diets: vitamin A, 9000 IU; vitamin D_3, 3000 IU; vitamin E, 24 IU; vitamin K_3, 3 mg; vitamin B_1, 3 mg; vitamin B_2, 7.5 mg; vitamin B_6, 3.6 mg; vitamin B_{12}, 0.36 mg; biotin, 1.5 mg; pantothenic acid, 15 mg; niacin, 30 mg; folic acid 1.5 mg. [2] Mineral premix provided following per kg of diets: Fe ($FeSO_4·H_2O$) 100.0 mg; Cu ($CuSO_4·5H_2O$) 5.0 mg; Zn ($ZnSO_4·H_2O$) 80.0 mg; Mn ($MnSO_4·H_2O$) 3.0 mg; I (KI) 0.14 mg; Se(Na_2SeO_3) 0.25 mg. [3] Measured values. RSM, rapeseed meal; ERM, extruded rapeseed meal; EERM, extruded solid-state enzymatically hydrolyzed rapeseed meal; CSD, corn-soybean meal diet; RSD I, rapeseed meal diet I; ERSD I, extruded rapeseed meal diet I; EERSD I, extruded solid-state enzymatically hydrolyzed rapeseed meal I.

Table S3. Ingredient composition and nutritional level of phase Ⅱ (as-fed basis, %)

Ingredients	NFD	RSD II	ERSD II	EERSD II
Ingredient composition				
Corn starch	80.54	54.54	54.54	54.54

RSM	0.00	30.00	0.00	0.00
ERM	0.00	0.00	30.00	0.00
EERM	0.00	0.00	0.00	30.00
Soybean oil	2.00	2.00	2.00	2.00
Sucrose	10.00	10.00	10.00	10.00
Cellulose	4.00	0.00	0.00	0.00
Choline chloride (50%)	0.15	0.15	0.15	0.15
$CaCO_3$	0.18	0.18	0.18	0.18
$CaHPO_4$	1.90	1.90	1.90	1.90
NaCl	0.50	0.50	0.50	0.50
Vitamin premix[1]	0.03	0.03	0.03	0.03
Mineral premix[2]	0.30	0.30	0.30	0.30
Cr_2O_3	0.40	0.40	0.40	0.40
Total	100.00	100.00	100.00	100.00
Analyzed composition[3]				
Dry matter	90.16	89.56	89.55	90.67
Gross energy, MJ/kg	15.03	15.54	15.64	15.64
Crude protein	0.57	11.32	12.57	11.51
Essential AA				
Lys	0.01	0.55	0.45	0.43
Met	0.00	0.15	0.11	0.11
Thr	0.01	0.48	0.40	0.43
Trp	0.00	0.13	0.11	0.12
Val	0.01	0.59	0.51	0.54
Ile	0.00	0.48	0.41	0.44
Leu	0.02	0.81	0.71	0.75
Phe	0.01	0.54	0.48	0.51
His	0.00	0.36	0.32	0.33
Arg	0.01	0.54	0.45	0.44
Non-essential AA				
Asp	0.02	0.75	0.64	0.69
Ser	0.01	0.44	0.34	0.37
Glu	0.03	2.15	1.90	2.01
Gly	0.01	0.55	0.48	0.51
Ala	0.00	0.48	0.41	0.45
Cys	0.01	0.13	0.12	0.12
Tyr	0.01	0.25	0.25	0.26
Pro	0.02	0.72	0.68	0.77
TAA	0.18	9.95	8.62	9.13

[1] Vitamin premix provided the following per kg of diets: vitamin A, 9000 IU; vitamin D_3, 3000 IU; vitamin E, 24 IU; vitamin K_3, 3 mg; vitamin B_1, 3 mg; vitamin B_2, 7.5 mg; vitamin B_6, 3.6 mg; vitamin B_{12}, 0.36 mg; biotin, 1.5 mg; pantothenic acid, 15 mg; niacin, 30 mg; folic acid 1.5 mg. [2] Mineral premix provided following per kg of diets: Fe ($FeSO_4 \cdot H_2O$) 100.0 mg; Cu ($CuSO_4 \cdot 5H_2O$) 5.0 mg; Zn ($ZnSO_4 \cdot H_2O$) 80.0 mg; Mn ($MnSO_4 \cdot H_2O$) 3.0 mg; I (KI) 0.14 mg; Se(Na_2SeO_3) 0.25 mg. [3] Measured values. RSM, rapeseed meal; ERM, extruded rapeseed meal; EERM, extruded solid-state enzymatically hydrolyzed rapeseed meal; NFD, N-free diet; RSD II, rapeseed meal diet II; ERSD II, extruded rapeseed meal diet II; EERSD II, extruded solid-state enzymatically hydrolyzed rapeseed meal II; AA, Amino acid; TAA, Total amino acid.
